# Successful Midwifery—Baby Incubator

**Published:** 1882-11

**Authors:** 


### SUCCESSFUL MIDWIFERY—BABY INCUBATOR.

The most successful results ever obtained in the Maternité Hospital in Paris (a mortality of only ¾ of one p. c.) have been reached in the new pavilion, of which M. Tarnier says : Each patient there has a separate room, entered from without, so that a nurse can only pass from one to another by going outside into the open air. The furniture is of japanned iron ; the floors, walks, and ceilings of impermeable concrete. The mattresses and pillows are stuffed with cut chaff, which is burnt after use in every single case. For the McIntosh sheet is substituted one of brown paper, made impermeable by pitch ; this is burnt after use. For the washing of the genitals weak solutions of bichloride of mercury are employed as being the best and most powerful germicide. The same enthusiastic accoucher has invented a sort of incubator in which babies of an age heretofore considered non-viable are placed. This machine consists of two compartments, the lower containing hot water to furnish the heat, and the upper for the reception of the child, where he is enveloped in cotton and under a glass tube. The results are said to be very encouraging.—*Bost. Med. and Surg. Journ.*

